# Concordance between the original and short version of the Impulsive Behaviour Scale UPPS-P using an IRT model

**DOI:** 10.1371/journal.pone.0194390

**Published:** 2018-03-21

**Authors:** Óscar M. Lozano, Carmen Díaz-Batanero, Antonio J. Rojas, Angelina Pilatti, Fermín Fernández-Calderón

**Affiliations:** 1 Department of Clinical and Experimental Psychology, University of Huelva, Campus de «El Carmen», Avenida de las Fuerzas Armadas, Huelva, Spain; 2 Psychology Department, University of Almeria, La Cañada de San Urbano, Almería, Spain; 3 National University of Córdoba, Ciudad Universitaria, Enfermera Gordillo Córdoba, Argentina; Deakin University, AUSTRALIA

## Abstract

The UPPS Impulsive Behaviour Scale (with five dimensions of impulsive behaviour) is being widely used. One of the handicaps of this instrument is its relatively long administration time. This has led to the development of a short version: SUPPS-P. There are no studies comparing the relationship between the SUPPS-P scores and the original UPPS-P scores. The objectives of this study, therefore, were to analyse the psychometric properties, concordance of person measures, and efficiency of the SUPPS-P compared to those on UPPS-P, applying an Item Response Theory Model. The UPPS-P and SUPPS-P were administered to 455 undergraduate students. Confirmatory factorial analysis replicated structures reported in previous studies: the five correlated factors structure and the model with two second-order factors (Urgency and Lack of Awareness) with Sensation Seeking dimension as a different factor. Rasch analysis show that both of the instruments presented adequate model-data fit. The results show the measurement for each dimension of UPPS-P offered more precision than SUPPS-P. The structure of items location was maintained in each dimension of SUPPS-P compared to the UPPS-P, but with better person and item separation indices of the UPPS-P dimensions. The concordance analysis reveals high correlations values between scores on both versions. From the standpoint of reducing items, it can be considered that the reduced version is more efficient. This study does not support the equivalence of items on the dimensions of Sensation Seeking and Lack of perseverance.

## Introduction

Impulsivity is a multidimensional construct characterized by the presence of behaviours without sufficient deliberation about the consequences, and the lack of behavioural inhibition [1]. It is present in different mental disorders, such as personality, attention deficit and hyperactivity or substance abuse disorders, among others [2]. It has also been found that impulsive behaviours affect healthy people in terms of their personal development, productivity and health [3].

Measuring this construct is a complex task, and has been typically distinguished using two types of instruments: using self-report measures (such as the Eysenk Impulsiveness Inventory [4], the Barratt Impulsiveness Scale [5] or the UPPS Impulsive Behaviour Scale [6]); and using impulsive behaviour tasks (ie. the Iowa Gambling Task [7], Stop Signal Task [8] or the Go/No Go [9] task). Sharma, Markon, and Clark [10] found in a meta-analysis study that when using self-report measures it is possible to distinguish four impulsivity traits: Sensation Seeking, Negative Urgency, lack of Planning, and lack of Perseverance. Similarly, with the behavioural tasks they established four factors labelled as Inattention, Inhibition, impulsive Decision-Making, and Shifting. These authors suggest that, despite the fact that the relationship between these two types of measurements is low, there are methodological and theoretical reasons to suggest that both types are related.

Among the self-report scales, UPPS evaluates the factors previously described in the meta-analysis of self-report impulsivity measures [10]. This scale emerged from a review conducted by Whiteside and Lynam [6], in which they analysed the dimensions shared between the different tests measuring impulsivity. They proposed a scale including the following dimensions or facets of impulsive behaviour: Urgency (defined as the tendency to be involved in risky behaviours in conditions of negative affectivity regardless of the negative consequences it could imply), (Lack of) Premeditation (difficulty to think and thinking about the consequences of a given behaviour before doing it), (Lack of) Perseverance (which refers to the inability to focus on executing a given task that could be difficult or boring), and Sensation Seeking(the tendency to enjoy activities with a high emotional component and the ability to open up to new experiences that could be dangerous) [11]. Subsequent work showed that the inclusion of Positive Urgency (the tendency to engage in actions whilst being in an unusually positive mood) dimension improve the construct validity [12], [13], resulting the UPPS-P scale.

In general, the theoretical consistency of the UPPS-P for measuring different factors of impulsivity, its usefulness in the study of relationships with psychopathological disorders, disruptive and risk behaviours, and the adequate psychometric properties previously described have made this scale one of the most widely used in recent years. Berg, Latzman, Bliwise & Lilienfeld [14] found 277 articles published in the PsycINFO database using this instrument. In Google Scholar this instrument appears referenced in more than 600 articles from its initial publication up to the present. It has been adapted into French [15], Spanish [16] or German [17]. All previous adaptation studies have shown adequate reliability coefficients and favourable evidence of validity based on both the internal structure of the scale and the relation with other variables.

One of the handicaps of this instrument is its relatively long administration time. The 59 items takes around 15 minutes to be administered. This limits its usefulness in some clinical and research settings, where a comprehensive evaluation of patients need the administration of several different tests. Therefore, efficient instruments in terms of information obtained according to the time spent are required [18]. This has led to the development of a reduced version of the UPPS-P. The short version (herein after SUPPS-P) was developed by Billieux et al. [19]. These authors proposed a version with four items in each dimension of impulsive behaviour. Items selected were those with higher factor loadings in each dimension. The empirical study showed adequate internal consistency reliability for each dimension (Cronbach’s alpha ranged from .70 to .84 in each dimension) and test-retest correlations ranging from .84 to .92. Confirmatory factor analysis supported two main factorial structures: 1) a model with the five inter-correlated dimensions; and, 2) another model with a hierarchical factorial structure with two second-order factors: Urgency (which includes the dimensions of Positive and Negative Urgency) and Lack of Awareness (including Lack of Premeditation and Lack of perseverance), whilst the Sensation Seeking dimension constituted a different factor.

From this SUPPS-P, the Spanish [20] and Italian [21] versions were developed. In both versions an adequate internal consistency reliability was obtained: in the Spanish version, the Cronbach’ alpha coefficient ranged from .61 to .81, and in the Italian version ranged from .73 to .84.

In terms of its factorial structure, in the Spanish version an adequate fit to the five factor model was found, whereas the hierarchical structure with two second-order factors analysed by Billieux et al. [19] was not supported. In the Italian version, the data was consistent with the five correlated dimensions structure, as well as with the hierarchical structure. However, the authors of this version compared both structures, and obtained a more favourable fit for the five correlated dimensions model.

Furthermore, Lynam [22] developed another English short version of the UPPS-P. Instead of the item selection process used by Billieux at al. [19], this author selected the items with higher total item correlations on each dimension. In terms of the factorial structure, the results provided by Cyders et al. [23] suggest that the data fit to both factorial models.

Although the SUPPS-P has been adapted to different languages, there are no studies comparing the relationship between the SUPPS-P scores and the original UPPS-P scores. According to the Standards for Educational and Psychological Measurement Testing [24], when comparing two versions of a scale it is necessary to provide evidence of score comparability. Reducing items can affect the equivalence of the measured construct, which can lead to errors in the interpretation of the scores [25].

Some authors note that Item Response Theory (IRT) provides an appropriate psychometric framework for studying measurement equivalence [26]. In addition to the advantages of using IRT models [27], these models are suitable to study the concordance between scores obtained on scales and their reduced versions [28], [29]. In addition, IRT models have useful metric properties for the development of reduced versions [30], [31], whenever an adequate fit between the model and data is found.

Considering the theoretical conceptualisation of the UPPS-P, its wide applicability, and the need to confirm the concordance between this and the reduced version, the objectives of this study were to: a) compare the factorial structure of the SUPPS-P with the items proposed by Lynam [22] and those proposed by Billieux et al. [19], b) to analyse the psychometric properties of the SUPPS-P compared to the UPPS-P, applying a model based on the IRT; and, c) to provide a comparative analysis of the concordance and efficiency of scores obtained by participants on the SUPPS-P and the UPPS-P.

## Materials and method

### Participants

The sample consists of 455 undergraduate students from the degree in Psychology and Early Childhood Teaching. Of these, 8.4% students belong to University of Granada, 13.4% to University of Cadiz, 16% to University of Almeria, and 62.2% to University of Huelva. The age of participants ranged from 18 to 57 years, with the average value in 21.52 years (SD = 5.15) and 80.1% are women.

### Instruments

The Spanish version of the UPPS-P adapted by Verdejo-García et al. [16] was administered. This scale consists of 59 items assessing the following dimensions [11], [12]: i) Negative Urgency (12 items) defined as the tendency to behave rashly in response to negative emotions; ii) Positive Urgency (14 items) defined as the tendency to lose control over their behavior when experiencing positive emotions; iii) Lack of Premeditation (11 items) refers to a difficulty in thinking and reflecting on the consequences of an act before engaging in that act, and it is considered as a prototypical element of impulse control; iv) Lack of perseverance (10 items) refers to a person inability to remain focused on a task that may be difficult or boring; and, v) Sensation Seeking (12 items) related to the tendency to enjoy exciting activities, and openness to try new experiences that may be dangerous.

UPPS-P items have a Likert format with five alternatives: from “1” (strongly agree) to “5” (strongly disagree). For each of the five dimensions of the UPPS-P, total scores were obtained. Higher scores in each dimension indicate greater impulsivity in these dimensions.

For analysing the items proposed by Lynam [22] and Billieux et al. [19] (on which the Spanish version is based) we used the 20 items specified by these authors.

### Procedure

The administration procedure of the UPPS-P was carried out along with other measures, including social-demographic questions (age, gender, and years of education), the Spanish version of the Substance Use Risk Profile Scale (SURPS) [32] and the AUDIT [33].The aim of the study was to obtain psychometric evidence related to impulsivity and drug consumption.

The test was administered by a member of the research team in group sessions in university classrooms, in groups of 38 to 45 students.

The objectives of the study were firstly explained and students were asked for voluntary participation. The students were also informed about the anonymity of the questionnaire and the duration of the test (between 30 and 40 minutes). They were also told that they were free to withdraw from the study at any time. Before starting the test, all the participants signed a consent form.

A total of 475 students participated in the study. Of these total, 20 were removed because of excessive missing data in UPPS items (more than 20%). This missing data might influence on the parameter estimations and consequently could have a possible effect in the analysis of the concordance.

No reward was granted to the students for their participation in the study.

This study was approved by the ethics committee of the University of Huelva.

### Statistical analysis

In order to contrast factorial structures reported in previous literature [19], [23], confirmatory factor analysis (CFA) was applied on the SUPPS-P. That is, two model were checked: (Model 1) with the five correlated dimensions, and (Model 2) with a factorial hierarchical structure with two second-order factors (Urgency and Lack of Awareness), and the Sensation Seeking dimension as a different factor. Each of these models was compared using the items proposed for the Billieux at al. [19] version and items proposed by Lynam [22].

For each of the analysis carried out, fit indices used were the CFI, NNFI (acceptable values > .90) and RMSEA (< .08 for an average value, and .08 for the interval higher than 95%) [34]. Given that there was multivariate non-normality, the maximum likelihood estimator with robust standard errors was used for analysis. The CFA analyses were carried out with EQS software, version 6.2.

The psychometric properties of items were analysed using a Rasch model for polytomous items, specifically the Rating Scale Model (RSM) [35]. RSM is a modelling procedure which involve fitting data to model, assuming that the probability of response to a category of an item is represented by a logistic function determined by person’s ability (known as person’s parameters–*θ*–which allows locating persons on the continuum, in this case according to his/her level of impulsivity) and the item difficulty (known as difficulty’s parameter–*β*–, which locate the items on the continuum, in this case according to how each item measures the impulsiveness trait). These models have unidimensionality among their assumptions, and we thus applied RSM to each of the dimensions of the UPPS independently. This model converts persons and items raw scores to interval measures which can be located on the same metric, transforming data to the “logit” scale, with mean 0 and standard deviation 1 [36].

In order to interpret the results obtained when applying the Rasch models, the fit of observed data to the Rasch model was firstly checked. This was carried out using residual analysis, which tests the degree to which the test response data are as expected from the model. For this analysis, the continuum is divided into *K* intervals, and the percentage of correct responses *P*_*jk*_ and the percentage of responses expected according to the model are evaluated for each interval *E(P*_*jk*_*)*. Rasch proposed the use of two chi-square statistics, reported as mean-square (MnSq), to interpret data fit: INFIT (sensitive to unexpected behaviour affecting responses to items near the person measure level) and OUTFIT (sensitive to unexpected behaviour by persons on items far from the person measure level).

MnSq values range from 0 to infinity. According to Linacre [37], values greater than 2 indicate aberrant response patterns that distort or degrade the measurement; values in the range of 1.5 and 2.0 are unproductive for measurement, but not degrading; values in the range of 0.50 to 1.50 are productive for measurement (good fit); and, values smaller than 0.5 indicate deterministic response patterns (they do not distort measurement but can lead to spurious high reliabilities).

The RSM uses a separation index for people and another for items instead of using reliability coefficients. Person Separation Index (PSI) represents the number of statistically different performance strata that the instrument can detect in the sample. Low PSI (<2) indicates that the instrument could be not sensitive to distinguish between people with high and low ability (in this case, impulsivity). Item Separation Index (ISI) is used to determine the number of strata of item impulsivity obtained in the scale. Values of ISI < 3 suggests that the person sample is not large enough to confirm the position of the items in the continuum [38].

Measurement precision has been estimated through the information function, which corresponds to the inverse of the standard error of estimation. Through this, the precision of items on each dimension of the original and the reduced version can be compared along the entire continuum.

All Rasch analysis were conducted with WINSTEPS software version 3.64.2 [39].

In order to check concordance between SUPPS-P and UPPS-P scores, a lineal regression model and a Reduction in Uncertainty index (RiU) [40] were estimated.

Reduction in uncertainty (RiU) can be used to decide whether to choose prediction or concordance to link two sets of scores [41]. Reduction in Uncertainty (RiU) is defined as RiU = 1 – √ (1 − *r*^2^) (where r is the correlation coefficient between both test scores) or RiU = 1- coefficient of alienation (which is a measure of statistical uncertainty about a dependent variable that remains after inclusion of information from the predictor variable). When *r* = 0, there is a 0% reduction; when *r* = 1, there is 100% reduction. For example, if the information in a short test has no relationship with variation in scores on the original test to be predicted, then the short test does nothing to reduce uncertainty about performance on the original test. It is reasonable to expect that at least 50% of uncertainty reduction in one score resulted from the other score [42]. If a predictor cannot decrease uncertainty by at least 50%, it is unlikely that it can operate as a valid surrogate, via concordance or equating, for the score being predicted [41].

The study of efficiency of the short version compared to the original was made through the formulation of Dennis, Chan and Funk [43], according to which

Efficency=(nºofshortscaleitemsnºoffullscaleitems)diagonalcorrelation

In the context of this study, a measure is more efficient in relation to another if provides the same information with a lower number of items. In accord with Dennis et al. [43], an efficiency criterion ≤ .80 was considered.

## Results

### Factorial structure of the SUPPS-P

The analysis of the correlations between the dimensions of the SUPPS-P with the items of the Billieux version [19] shows that all correlations are statistically significant (ranging from *r* = .144 to *r* = .476) except for the relationships between negative urgency and lack of perseverance (*r* = .084). The values of the correlations of the SUPPS-P following those proposed by Lynam [22] were statistically significant for all the cases (ranging between *r* = .161 to *r* = .548), except for the relationships between negative urgency and lack of perseverance (*r* = .069) and lack of perseverance and sensation seeking (*r* = .093). The values of the correlations between the dimensions of the SUPPS-P calculated with the items of the Billieux and Lynam versions [19], [22] ranged between *r* = .759 and *r* = .873.

The results of the fit analysis of the different models proposed for the factorial structure of the SUPPS-P, using the items of the Billieux version [19] and the items of the Lynam version [22] are shown in Table 1. Results show inadequate fit in both models, particularly for NNFI and CFI (i.e. < .90) when the items proposed by Lynam are used.

**Table 1:** Fit statistics of the factorial structure of the different models.

	S-B χ^2^/gl	NNFI	CFI	RMSEA (IC 95%)
Items of the Billieux version				
Model 1 (five correlated factors)	1.275	0.967	0.972	0.032 (0.016, 0.044)
Model 2 (three correlated factors, with two second-order factors)	1.288	0.967	0.971	0.032 (0.017, 0.044)
Items proposed by Lynam				
Model 1 (five correlated factors)	1.608	0.858	0.880	0.047 (0.036, 0.057)
Model 2 (three correlated factors, with two second-order factors)	1.579	0.864	0.884	0.046 (0.035, 0.056)

Consequently, the following analyses were carried out for each of the dimensions with the items proposed for the Billieux version.

### Fit between data and model

Fit statistics (MnSq-INFIT and MnSq-OUTFIT) of both the complete and short version are displayed in Table 2. The summary fit analysis showed acceptable standardized squared residual values on all dimensions for the UPPS-P and the SUPPS-P (Table 2). Regarding item fit indices, only item 57 on Positive Urgency dimension on UPPS-P showed residual mean-square values exceeding acceptable fit. All items of SUPPS-P presented adequate INFIT and OUTFIT values.

**Table 2:** Fit statistics for UPPS-P and SUPPS-P items.

Dimensions		UPPS-P				SUPPS-P			
Negative Urgency	Items	Measure (error)	MNSQ-INFIT	MNSQ-OUTFIT	Item-total correlation	Measure (error)	MNSQ-INFIT	MNSQ-OUTFIT	Item-total correlation
	Item 2	.064 (.07)	0.85	0.86	.61				
	Item 7	-0.30 (.06)	1.35	1.35	.51				
	Item 12	0.22 (.06)	1.30	1.29	.49				
	Item 17	0.81 (.07)	1.09	1.07	.53				
	Item 22	0.75 (.07)	1.21	1.21	.55				
	Item 29	-0.35 (.06)	0.79	0.78	.71	-0.34 (.09)	1.05	1.05	.80
	Item 34	0.06 (.06)	0.84	0.87	.67	0.43 (.09)	1.20	1.21	.77
	Item 39	-0.88 (.07)	1.37	1.39	.67				
	Item 44	0.30 (.07)	0.71	0.70	.76	0.87 (.09)	0.79	0.78	.86
	Item 50	-0.69 (.07)	0.79	0.80	.67	-0.96 (.09)	0.92	0.90	.81
	Item 53	-0.47 (.06)	0.97	1.01	.48				
	Item 58	-0.09 (.06)	0.75	0.75	.72				
	Summary of items (mean)	0.00 (0.07)	1.00	1.01		0.00 (0.09)	0.99	0.99	
	Summary of persons (mean)	-0.35 (0.45)	1.01	1.01		-0.26 (1.04)	1.00	0.99	
Lack of Premeditation	Item 1	-1.06 (.08)	1.33	1.39	.50				
	Item 6	-0.75 (.08)	1.07	1.09	.61	-1.21 (.09)	1.28	1.35	.71
	Item 11	-0.30 (.08)	1.50	1.51	.59				
	Item 16	0.63 (.08)	0.83	0.84	.67				
	Item 21	-0.07 (.08)	1.33	1.39	.51				
	Item 28	0.83 (.09)	0.87	0.86	.62				
	Item 33	-0.02 (.08)	0.79	0.80	.73	-0.11 (.10)	0.83	0.84	.82
	Item 38	-0.01 (.08)	0.70	0.70	.70				
	Item 43	0.90 (.09)	0.88	0.88	.61	1.24 (.10)	1.11	1.12	.68
	Item 48	0.11 (.08)	0.60	0.61	.78	0.08 (.10)	0.73	0.73	.82
	Item 55	-0.26 (.08)	0.99	0.98	.67				
	Summary of items (mean)	0.00 (0.08)	0.99	1.00		0.00 (0.10)	0.99	1.01	
	Summary of persons (mean)	-1.16 (0.59)	1.02	1.00		-1.41 (1.16)	1.01	1.01	
Lack of Perserverance	Item 4	1.29 (.09)	1.08	1.08	.49	1.33 (.12)	1.44	1.41	.65
	Item 9	0.50 (.08)	1.40	1.36	.49				
	Item 14	0.01 (.07)	1.02	0.99	.58				
	Item 19	-0.76 (.07)	1.07	1.13	.45				
	Item 24	-1.37 (.07)	1.08	1.11	.51				
	Item 27	0.49 (.08)	0.61	0.61	.69	-0.14 (.11)	0.83	0.82	.81
	Item 32	-0.21 (.07)	1.06	1.05	.54				
	Item 37	0.12 (.08)	0.69	0.70	.66	-0.90 (.11)	1.03	1.02	.78
	Item 42	0.42 (.08)	0.56	0.58	.69	-0.29 (.11)	0.68	0.67	.83
	Item 47	-0.49 (.07)	1.44	1.42	.56				
	Summary of items (mean)	0.00 (0.08)	1.00	1.00		0.00 (0.12)	1.00	0.98	
	Summary of persons (mean)	1.27 (0.58)	1.02	1.00		-2.65 (1.22)	0.98	0.98	
Sensation Seeking	Item 3	-0.49 (.06)	0.72	0.84	.58	-1.33 (.09)	1.29	1.40	.70
	Item 8	-0.01 (.06)	1.21	1.30	.45				
	Item 13	0.05 (.06)	1.18	1.33	.42				
	Item 18	-0.63 (.06)	1.18	1.20	.61				
	Item 23	0.78 (.06)	0.73	0.75	.67	1.56 (.09)	0.98	0.94	.81
	Item 26	-0.09 (.06)	1.27	1.19	.66				
	Item 31	-0.09 (.06)	0.52	0.53	.76	-0.41 (.09)	0.78	0.77	.86
	Item 36	0.45 (.06)	1.28	1.24	.61				
	Item 41	0.17 (.06)	0.63	0.65	.70	0.18 (.09)	0.89	0.85	.83
	Item 46	0.21 (.06)	0.82	0.79	.72				
	Item 51	-0.26 (.06)	1.27	1.22	.64				
	Item 56	-0.09 (.06)	1.26	1.25	.58				
	Summary of items (mean)	0.00 (0.06)	1.01	1.02		0.00 (0.09)	0.99	0.99	0.00 (0.06)
	Summary of persons (mean)	0.12 (0.41)	1.02	1.02		0.04 (1.08)	0.98	0.99	0.12 (0.41)
Positive Urgency	Item 5	-0.16 (.08)	1.07	1.11	.68				
	Item 10	0.62 (.09)	1.00	1.14	.65				
	Item 15	0.86 (.09)	0.78	0.73	.69				
	Item 20	0.25 (.09)	0.89	0.83	.71				
	Item 25	0.09 (.08)	1.06	1.08	.66				
	Item 30	0.36 (.09)	0.82	0.76	.71				
	Item 35	1.49 (.10)	1.11	0.94	.57				
	Item 40	0.74 (.09)	0.72	0.61	.72	1.30 (.11)	1.13	1.11	.75
	Item 45	-0.69 (.08)	1.15	1.11	.71	-0.94 (.10)	1.18	1.19	.83
	Item 49	-0.21 (.08)	0.83	0.82	.73	-0.17 (.10)	0.89	0.84	.84
	Item 52	-0.23 (.08)	0.69	0.71	.76	-0.20 (.10)	0.77	0.78	.86
	Item 54	0.06 (.08)	1.05	0.99	.67				
	Item 57	-1.93 (.08)	1.54	1.84	.56				
	Item 59	-1.23 (.08)	1.33	1.40	.64				
	Summary of items (mean)	0.00 (0.09)	1.00	1.00		0.00 (0.11)	0.99	0.99	
	Summary of persons (mean)	-1.77 (0.56)	1.04	1.00		-1.59 (1.13)	0.99	0.98	

*Note*: MNSQ: Mean Square Residual.

### Separation and information function

PSI on UPPS-P indicate that items on dimensions of Positive Urgency, Lack of Premeditation, Negative Urgency and Sensation Seeking allow discrimination between persons with low and high levels on each dimension (Table 3). However, on the Lack of Perseverance dimension, lower than recommended values [38] were observed. On the reduced version, this coefficient values are adequate when computed on the dimensions of Positive Urgency and Sensation Seeking. On the other dimensions the value obtained is lower than recommended (<2). Regarding ISI, appropriate values were obtained for both versions.

**Table 3:** Separation measures of dimensions on each version.

		Persons separation	Items separation
Negative Urgency	UPPS-P	2.37	8.11
	SUPPS-P	2.09	7.90
Lack of perseverance	UPPS-P	1.87	9.33
	SUPPS-P	1.48	7.28
Lack of Premeditation	UPPS-P	2.47	7.21
	SUPPS-P	1.72	8.77
Sensation Seeking	UPPS-P	2.42	6.05
	SUPPS-P	2.11	11.52
Positive Urgency	UPPS-P	2.93	9.84
	SUPPS-P	1.79	7.74

Note that from Table 3 it can be observed that PSI are lower on UPPS-P compared to SUPPS-P. On ISI, no notable differences were observed between the versions.

**Fig 1:**
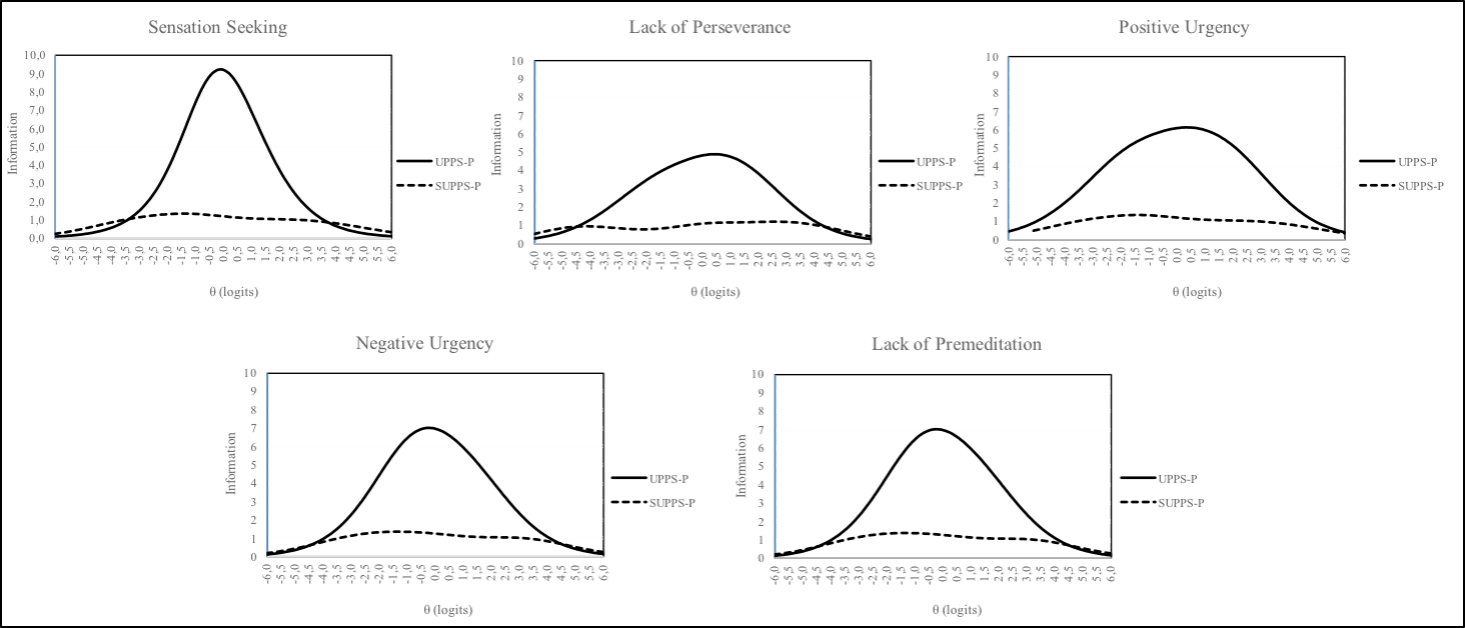
Information functions of UPPS-P and short version (SUPPS-P). X-Axis: (θ-logits) Level of ability; Y-Axis: Amount of information.

With respect to the precision of the measure, Fig 1 shows information functions of each dimension. It is clear that the information functions of the dimensions of the UPPS-P are higher than the information functions of the UPPS.P. This indicates that when using the UPPS-P we obtain a more precise person’s measure of each dimension than using the SUPPS-P.

### Items map

**Fig 2:**
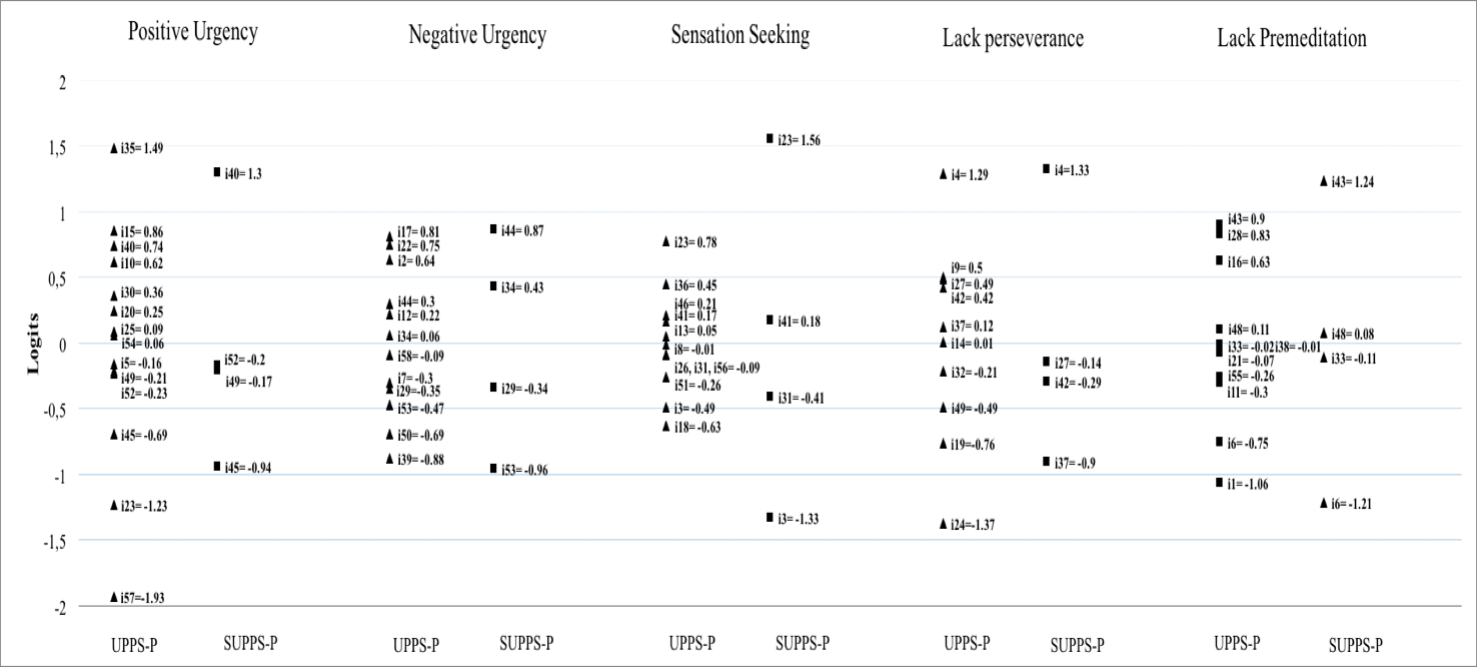
Item map of short and original versions. Note: Fig 2 represents the distribution of each item (i#) of the UPPS-P and SUPPS-P by its “difficulty” parameter–β- (location of items at the continuum). X-Axis: UPPS-P and SUPPS-P items; Y-Axis: “difficulty” parameter of items (in logits); i#: item number.

Item distribution according to their difficulty parameters on each dimension are presented on Fig 2. It can be seen that the structure of items location is maintained in each dimension of SUPPS-P compared to the UPPS-P. That is, the item difficulty parameters of each version maintain their order on all dimensions. It is appreciable that the distance between items in the reduced version is significantly higher than that the distance observed between items of UPPS-P. This distance between items reaches 1.5 logits in the case of Positive Urgency dimension. In contrast, the items on UPPS-P dimensions are generally closer. However, in this version there are also some items with notable distances respect to its adjacent, such as between items 57 and 45 of Positive Urgency dimension.

### Correlation between dimensions, concordance and reduction efficiency

**Fig 3:**
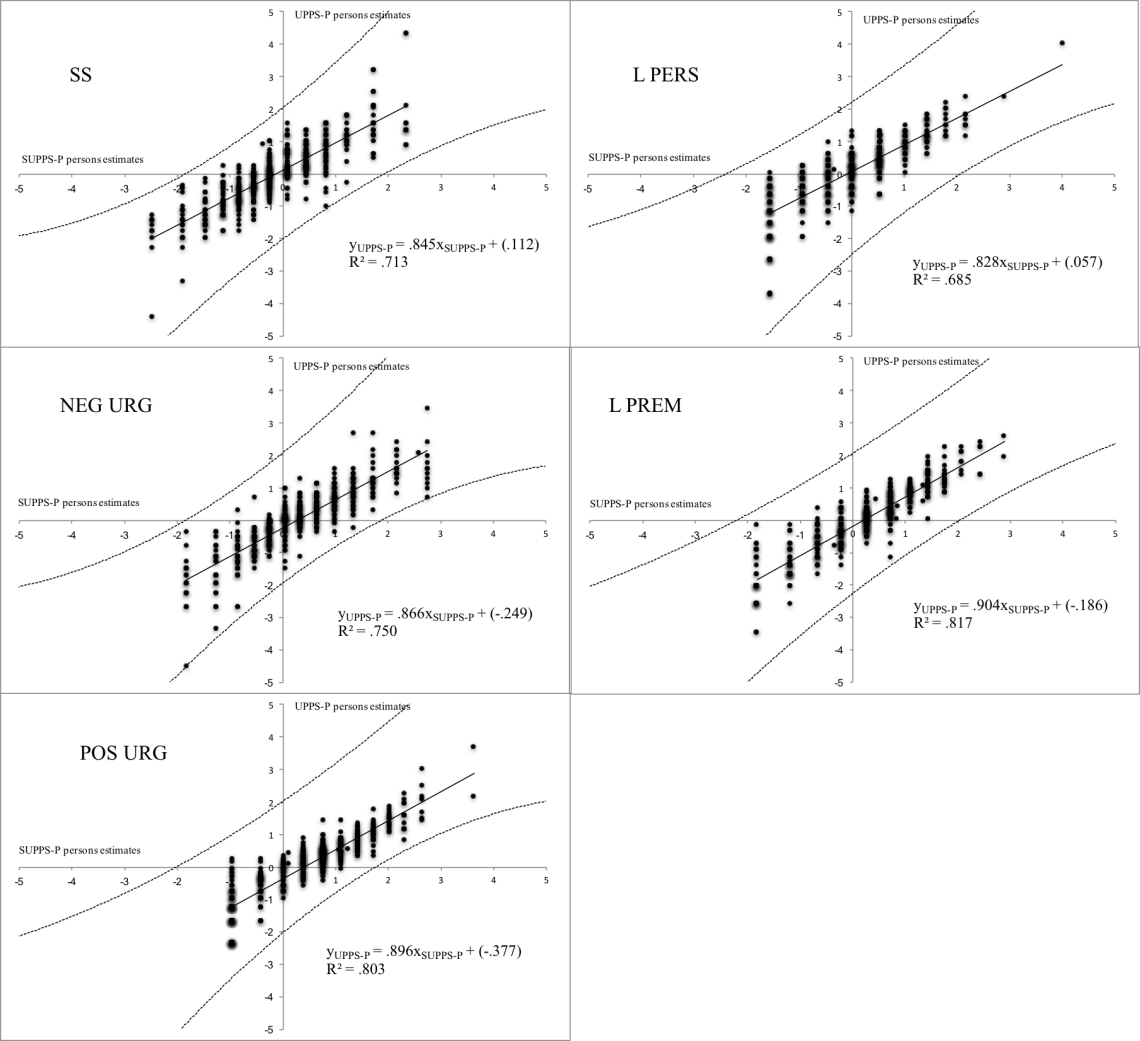
Concordance between original UPPS and short version (SUPPS-P). X-axis: z scores of SUPPS-P dimensions; Y-axis: z scores of UPPS-P dimensions. Dimensions: SS: Sensation Seeking; L PERS: Lack of Perseverance; NEG URG: Negative Urgency; L PREM: Lack of Premeditation; POS URG: Positive Urgency.

Fig 3 displays scatterplots of person scores in each dimension, estimated with the RSM. These graphics present, along with a regression line, two dotted lines representing the confidence intervals. Under ideal circumstances, the empirical regression line should have a slope of 1 and an intercept of 0. It can be noted in all figures that standardized slope values are between *β* = .828 (Lack of Perseverance) and *β* = .904 (Lack of Premeditation). The values estimated for the intercept ranged from -.377 to .112. R^2^ values oscillated between .685 (Lack of Premeditation) and 0.817 (Lack of Perseverance).

With regard to the concordance between versions, the data supported concordance with the full version on the dimensions of Negative Urgency (RiU = .500), Positive Urgency (RiU = .556), and Lack of Premeditation (RiU = .572). By contrast, the dimension of Lack of perseverance and Sensation Seeking the RiU values obtained were below .5. For these dimensions, concordance cannot be assumed.

In terms of efficiency of the reduced version, all dimensions present values below the criterion of ≤.80 recommended by Conrad et al. [24]. Thus, from the standpoint of reducing items, it appears that the reduced version is more efficient.

## Discussion

It is common to develop a reduced version of a scale when it requires a long administration time, as is the case of the UPPS-P. In such cases, the Standards of AERA, APA & NCME [24] states that it is necessary to test the equivalence between scores of different versions. To date, there has not been a published psychometric study analysing the concordance of the UPPS-P and its reduced version. Thus, the aim of the present paper was to provide such psychometric evidence, using IRT models. This study began by comparing, with a Spanish sample, the factorial structure of the SUPPS-P with the items of Billieux included in the Spanish version [20] and with the items proposed by Lynam [22] for the English version. Factorial structures checked included two models that had previous empirical support: a model with the five correlated dimensions, and a hierarchical model with two second-order factors. As shown in our study, the factorial structure of the SUPPS-P with the items proposed by Lynam [22] showed inadequate fit, which means lack of validity evidence for the SUPSS-P Spanish version with these items. By using the items of the Billieux version [19], adequate fit to both factorial models were found. These results are consistent with the evidence provided by Billieux et al. [19], D’Orta et al. [21] and Cyders et al. [23]. Our results are also consistent with those provided by Cándido et al. [20] regarding the factorial structure of the five correlated dimensions. However, those authors could not confirm the factorial structure with second-order factors.

Further, the application of the RSM reveal an adequate fit of the SUPPS-P items to the model. This permits interpretation of the scores and drawing of conclusions based on the results provided by this model. In this regard, one of the elements analysed was the location of the items on each of the dimensions. The results show that in all dimensions of the UPPS-P, items are well distributed along the continuum, with appropriate distances between the most adjacent items. However, in the short version it can be seen that some items are very close to each other: items 52 and 49 on Positive Urgency dimension, items 27 and 42 on Lack of Perseverance, and items 48 and 33 on Lack of Premeditation. When analysing the content included in these items, redundant wording can be found. For example, while item 27 states *'I finish what I start*', item 42 includes the sentence *'I almost always end projects I start*'; the very similar content on both items may be causing similar scale values on the continuum. In addition, in all dimensions except Negative Urgency, substantial distances are observed between adjacent items. According to Lai and Eton [44] a difference between adjacent items of 0.5 is considered to be a substantial distance. However, on items occupying central positions in the continuum, a distance greater than 0.3 could not detect clinically relevant differences in measurements. It is for this reason that with the SUPPS-P, some continuum regions should be covered. This visual detection of the 'holes' on the continuum is noticeable studying the separation indices. The PSI are found to be less than recommended on Positive Urgency, Lack of Perseverance and Lack of Premeditation. As a result, these dimensions may not be sensitive to differentiate between people with high and low levels of these dimensions. In such cases Linacre [39] points out some solutions to improve this coefficient, such as: increasing the number of items, analysing whether replacing the existing items by others of the same dimension produces an improvement, or increasing the number of response categories. Any of these measures will require a detailed study to find the most efficient solution.

Complementing this coefficient, the person standard error turned out to be lower in the UPPS-P than in the SUPPS-P. In particular, the standard error values for measurements of the short version are double than those values observed for the UPPS-P; that is, person measures with the short version has a greater error than those used in the original version, and hence they are measured with lower precision. Furthermore, as shown in the information function of each dimension, measurement using the items of UPPS-P is more accurate across the entire continuum.

Although SUPPS-P seems to obtain less accurate measures, the concordance analysis reveals high correlations values between scores on both versions. Our analyses have also revealed concordance between the scores of the dimensions of Positive Urgency, Negative Urgency, and Lack of Premeditation, with acceptable values of RiU index. However, on the dimensions of Sensation Seeking and Lack of Perseverance, RiU values do not reduce uncertainty below 50%. It is therefore unlikely that on these dimensions, the short version scores can be used as an adequate predictor of the original version^41^. However, the results have indeed shown that the short version is efficient on the other three dimensions.

The current paper provides evidence concerning the consistency of the original and reduced version of the UPPS-P, whilst new psychometric properties were also explored. However, it is necessary to consider some limitations of this work, which are derived from the sample used. Firstly, we should mention that the psychometric study is restricted to the Spanish version. Psychometric studies carried out with versions in English, French, or Italian are therefore necessary in order to determine the concordance of the scores between different versions in those languages. For the analysis, we used a sample of university students from four different universities. To a large extent, this group cannot be considered representative of the whole population. We should also take into account that in this study, the majority of the participants were female. However, other previous psychometric studies with the original and reduced version have also been developed with samples of university students [16], [19]-[21], [45], and then compared with other population groups [17]. In this regard, future studies should address whether concordance between scores on the observed dimensions can also be obtained in clinical samples. On the other hand, there were 20 participants who were removed due to excessive missing data in UPPS-P items. Previous analyzes showed that there were no statistically significant differences between these participants and the rest of the sample neither sociodemographic variables nor variables related to drug use. Bearing in mind that the aim of this study is to analyze the concordance between the versions, the authors considered more suitable to removed them rather than using a missing-data imputation method.

As a conclusion, we understand that in clinical settings, which usually requires a precise person measures, it may be the most appropriate the use of the UPPS-P, since it measures with more precision. However, in correlational research studies, the use of SUPPS-P could be justified by the high concordance between the scores of both versions. Nonetheless, it is necessary to draw attention to the fact that this study does not support the concordance of items on Sensation Seeking and Lack of Perseverance dimensions.

## Supporting information

S1 File Data file with measures reported. (ZIP)
